# Supplementary material for: Astrometrically registered maps of H2O and SiO masers toward VX Sagittarii
Source: Nat Commun. 2018 Jun 28;9:2534. doi: 10.1038/s41467-018-04767-8 (PMC6023886; doi:10.1038/s41467-018-04767-8)
Supplement: Supplementary file 1 — Supplementary Information [file 41467_2018_4767_MOESM1_ESM.docx]

Supplementary Fig. 1 │Another astrometrically registered integrated intensity maps of 22.2 GHz H_2_O and 43.1/42.8/86.2 GHz SiO masers obtained from SFPR toward VX Sgr. The rms noise levels on the maps are 62.96, 17.80, 9.58 and 85.63 Jy beam^-1^ m s^-1^ in order of the maser transition respectively. The peak flux values are 555.03, 64.33, 49.20 and 57.74 Jy beam^-1^ km s^-1^. The contour levels are adopted at 10 12 15 20 25 30 50· · · for multiples of each rms value.

Supplementary Fig. 2 │The velocity–position spot maps and flux spectra The color of the spot indicates the velocity and the size of spot the logarithmic scale of the intensity. The dashed circle is the ring fitted to the SiO maser spot distribution. The center of each ring is marked with '+'. The position of the central star is marked with 'X' on the H_2_O distribution and assumed to be the ring center of all three SiO maser lines combined in Supplementary Fig. 3. The spectra represent the single-dish (solid, February 15, 2016) spectra, and total power (dotted) and recovered flux (dashed) in VLBI observations. The gray vertical line represents the stellar velocity (5.3 km s^-1^).

Supplementary Fig. 3 │ Registered maser spot map of the SiO maser lines and their spot distribution histograms. The blue, red and yellow color indicates 43.1, 42.8 and 86.2 GHz SiO masers, respectively. The dotted circle shows the result of ring fitting using all the maser spot distributions of the three maser lines. The mark “x” indicates the ring fitting center assumed to be the position of the central star. Histograms represent the radial distribution of the maser spots with respect to the central star. The ring radius and FWHM derived from the Gaussian fitting are represented in each panel.

Supplementary Fig. 4 │ The velocity-position maps of the 129.3 GHz SiO single-band maser. The color of the spot indicates the velocity, and the size of the spot indicates the intensity of the log scale. The dashed rings indicate the ring fitting results at each epoch given in Supplementary Table 4, and the dotted rings are the same size as the 129.3 GHz maser on March 27, 2016 (φ=0.67) given in Table 1.

Supplementary Table 1 | Observation summary on March 27, 2016 (φ=0.67)

| Transition | Synthesized beam size*  (mas) | Position angle  (deg) | System noise temperature  (K) |
| --- | --- | --- | --- |
| H_2_O 6_16_-5_23_  (22.2 GHz) | 5.70 * 4.80 | -57.74 | ~150 |
| SiO v=1, J=1-0  (43.1 GHz) | 3.00 * 2.57 | -34.01 | ~200 |
| SiO v=2, J=1-0  (42.8 GHz) | 3.09 * 2.56 | -36.38 | ~200 |
| SiO v=1, J=2-1  (86.2 GHz) | 1.48 * 1.29 | -37.61 | ~300 |
| SiO v=1, J=3-2  (129.3 GHz) | 1.01 * 0.81 | -50.30 | ~500 |

* The uniform weighting was applied in the image processing

Supplementary Table 2 | SiO maser ring-fitting results on February 27, 2016 (φ = 0.63)

| SiO | R.A. offset* | Dec. offset* | Ring radius** | | Gaussian fit | Converted coordinate (J2000)*** | |
| --- | --- | --- | --- | --- | --- | --- | --- |
| Transition | (mas) | (mas) | (mas) | (AU) | (mas) | R.A. | Dec. |
| v=1, J=1–0 (43.1 GHz) | –60.146 | –25.947 | 13.78±0.49 | 21.63±0.77 | 13.68 | 18:08:04.0456685 | –22:13:26.625947 |
| v=2, J=1–0 (42.8 GHz) | –59.435 | –26.049 | 13.28±0.91 | 20.85±1.43 | 13.03 | 18:08:04.0457197 | –22:13:26.626049 |
| v=1, J=2–1 (86.2 GHz) | –60.326 | –25.858 | 14.09±0.60 | 22.12±0.94 | 13.86 | 18:08:04.0456555 | –22:13:26.625858 |
| Three SiO masers | –59.954 | –25.878 | 13.76±0.49 | 21.60±0.77 | … | 18:08:04.0456823 | –22:13:26.625878 |

*The offset value represents the position difference of the central star with respect to the observed Hipparcos coordinates.

**The astronomical unit (AU) was calculated using the distance of 1.57 kpc for VX Sgr.

***The coordinates are converted from the observed Hipparcos coordinates based on the R.A and Dec. offsets

Supplementary Table 3 | Recovering flux

| Transition | February 27, 2016 (φ=0.63) | | March 27, 2016 (φ=0.67) | |
| --- | --- | --- | --- | --- |
|  | VLBI total power  /single-dish flux | VLBI recovered  /total power flux | VLBI total power  /single-dish flux | VLBI recovered  /total power flux |
| H_2_O 6_16_-5_23_  (22.2 GHz) | 80.9% | 42.8% | 81.9% | 46.4% |
| SiO v=1, J=1-0  (43.1 GHz) | 61.9% | 48.1% | 77.3% | 43.5% |
| SiO v=2, J=1-0  (42.8 GHz) | 61.6% | 71.3% | 75.5% | 64.2% |
| SiO v=1, J=2-1  (86.2 GHz) | 69.4% | 29.8% | 68.3% | 37.5% |
| SiO v=1, J=3-2  (129.3 GHz) | … | … | 48.5% | 38.0% |

Supplementary Table 4 | SiO maser ring-fitting results of other four epochs with 129.3 GHz detected

|  | Ring radius* | | | | | | | |
| --- | --- | --- | --- | --- | --- | --- | --- | --- |
| SiO | 2015.03.14 ($\phi$=0.11) | | 2015.11.28 ($\phi$=0.50) | | 2015.12.30 ($\phi$=0.54) | | 2016.12.30 ($\phi$=1.10) | |
| Transition | (mas) | (AU) | (mas) | (AU) | (mas) | (AU) | (mas) | (AU) |
| v=1, J=1–0 (43.1 GHz) | 13.61±0.33 | 21.37±0.52 | 13.55±0.48 | 21.27±0.75 | 13.60±0.93 | 21.35±1.46 | 13.36±1.32 | 20.98±2.07 |
| v=2, J=1–0 (42.8 GHz) | 13.24±1.14 | 20.78±1.79 | 13.42±1.40 | 21.07±2.20 | 13.36±0.71 | 20.98±1.11 | 13.72±0.31 | 21.54±0.49 |
| v=1, J=2–1 (86.2 GHz) | No data | No data | 13.90±0.61 | 21.82±0.96 | 14.06±0.66 | 22.07±1.04 | 14.55±0.45 | 22.84±0.71 |
| v=1, J=3–2 (129.3 GHz) | 17.54±0.96 | 27.54±1.51 | 16.65±1.64 | 26.14±2.57 | 16.10±0.72 | 25.28±1.13 | 19.15±0.80 | 30.07±1.26 |

∗The astronomical unit (AU) was calculated using the distance of 1.57 kpc for VX Sgr
